# Mortality, risk factors and causes of death in Swedish patients with open tibial fractures - a nationwide study of 3, 777 patients

**DOI:** 10.1186/s13049-018-0531-0

**Published:** 2018-07-25

**Authors:** Ulrika Tampe, Lukas W. Widmer, Rüdiger J. Weiss, Karl-Åke Jansson

**Affiliations:** Department of Molecular Medicine and Surgery, Section of Orthopaedics and Sports Medicine, Karolinska Institutet at Karolinska University Hospital, Stockholm, Sweden

**Keywords:** Causes of death, Epidemiology, Mortality, Open fracture, Tibial fracture

## Abstract

**Background:**

Open tibial fractures are serious, complicated injuries. Previous studies suggested an increased risk of death, however, this has not been studied in large population-based settings. We aimed to analyze mortality including causes of death in all patients with open tibial fractures in Sweden. Moreover, we wanted to compare mortality rates with the Swedish population and determine whether treatment-related or demographic variables were independently associated with death.

**Method:**

We searched the Swedish National Hospital Discharge Register for all patients with open tibial fracture between 1998 and 2010. We collected the following variables: age, gender, length of stay, mechanism of injury and treatment rendered. We then cross-referenced the Swedish Cause of Death Register to determine the cause of death, if applicable. We compared mortality in the study population with population-based mortality data from Statistics Sweden and determined whether variables were independently associated with death using regression analysis.

**Results:**

Of the 3777 open tibial fractures, 425 (11.3%) patients died. The most common causes of death for elderly patients were cardiovascular and respiratory disease. Patients aged 15–39 years succumbed to external causes (accidents, suicides or poisoning). Increasing age (OR 25.7 (95% CI 11.8–64.8) *p* < 0.001), length of hospital stay (HR 1.01, (95% CI 1.01–1.02,) *p* < 0.001), limb amputation (OR 4.8 (95% CI 1.86–11.1) *p* < 0.001) and cause of the accident were independently associated with an increased mortality.

**Conclusion:**

Patients with open tibial fractures have an increased risk of death compared with the general population in all age- and gender-groups. External causes of death are over-represented and indicate a subgroup with a risky behaviour among younger males. Elderly patients have an increased risk of dying comparable to hip fracture patients. They are at risk for cardiovascular and respiratory failure and should be treated with urgency, emphasizing the need for specialized geriatric trauma units.

## Background

Open tibial fractures are serious injuries, which endanger both life and limb. The incidence in Northern Europe is between 2.3 and 3.4 per 100,000 person-years [1–3], and consists of a unimodal age distribution for males, whereas women have a more even age distribution. In fact, previous work identified the most common mechanisms of injury as motor vehicle accidents (43%), most commonly seen in males, age 15–50 years, and falls from a standing-height (21%), which are most commonly seen in females [2].

Treatment of open tibial fractures is difficult and fraught with complications. Problems such as osteomyelitis, non-union, mal-union, compartment syndrome and amputation contribute to an increase in healthcare costs (resource utilization) as well as a decrease in patient quality of life [4–7]. In fact, the amputation rate is reported to be 3.6% in a study involving adult Swedish patients [2]. However, in trauma centres dealing with a larger proportion of high-energy injuries, the amputation rate is much higher, about 25% [8, 9].

In contrast to the well-studied mortality rate after hip fractures [10, 11] few studies report the risk of death after open tibial fractures. It is possible that high complication rates translate to an increase in the risk of death. In fact, previous studies involving small populations of elderly patients demonstrated relatively high mortality after open tibial fractures [12, 13]. Other studies involving larger patient populations focus on closed as well as open fractures [14, 15]. However, neither provides a suitable population-based assessment of the risk of death following treatment for open tibial fracture.

As such, there are no population-based reports documenting short- and long-term mortality rates in patients with open tibial fractures. With this in mind, we aimed to analyse mortality rates, causes of death and risk factors for death in the Swedish population, with data from a nationwide inpatient register. In doing so, we describe the mortality after open tibial fracture in the Swedish population, compare the mortality rates with those in the general Swedish population, and determine whether treatment-related or demographic variables were independently associated with death.

## Methods

### Source of data

We obtained data from the Swedish National Hospital Discharge Register (SNHDR), where 98% of all hospital admissions in Sweden are covered [16]. The study period was 1998–2010. All Swedish individuals can be identified through a 10-digit national registration number and which allows epidemiological studies on a nationwide basis. Data on diagnosis, surgical procedure codes, and demographic data for each hospital admission in Sweden are available. Diagnoses are coded according to the International Classification of Diseases (ICD). We extracted data from the Register on all hospital admissions and re-admissions of patients (≥15 years of age) with the following diagnoses: open fractures of the proximal tibia (S82.11), tibial shaft (S82.21), and distal tibia (S82.31). No exclusions were made.

Mechanism of injury was collected from ICD E-codes and divided into categories: fall from standing height, fall from height, car accidents, transport accidents with unprotected road users (bicycle accidents, motor bike accidents, horse riders, and pedestrians hit by motor vehicles), self-destructive injuries and miscellaneous. Unprotected road users were gathered in one group as there were very few individuals in the sub-groups.

We cross-referenced data from the Swedish National Hospital Discharge Register to the Swedish Cause of Death Register [17, 18]. Dates of death and causes of death for patients from this cohort that died during the study period were extracted. Causes of death were coded according to the International Classification of Diseases (ICD 10) and grouped according to the European shortlist for causes of death [19]. According to this list, causes of death are grouped as diseases of the respiratory, circulatory, digestive, nervous and musculoskeletal system as well as malignant neoplasms, mental and behavioural disorders, endocrine and metabolic diseases, external, unknown and unspecified causes, and miscellaneous. External causes were further divided into transport accidents, accidental falls, accidental poisoning, suicide and intentional self-harm, homicide and assault, drowning accidents and events of undetermined intent.

Patients were grouped as young (15–39 years), middle-aged (40–64 years) and old (≥65 years). Males aged 15–60 years as a relatively large group was thoroughly investigated. We hypothesized that they would be more subjected to external causes and violent deaths, as shown in previous studies [20, 21].

The primary outcome variable was early mortality, defined as death within 90 days after injury. Furthermore, secondary outcomes were late mortality, defined as death between 90 days and 2 years, total mortality during the study period, length of stay (LOS), standard mortality ratios (SMR) and variables associated with death.

### Statistics

Logistic regression analysis was used to assess factors associated with death within 90 days, as prevalence of early death was not time-homogenous. Cox regression analysis was used for the correlative analysis from 90 days and beyond. We hypothesized that by doing this, we would estimate the most critical external causes directly associated with the earlier deaths. On the other hand, we would also analyse other long term factors that would influence the risk for a later death. Odds ratios (ORs), hazard ratios (HRs) and the associated 95% confidence intervals (CI) are presented. The crude results were adjusted for age, gender, mechanism of injury, occurrence of amputation, and length of stay.

The standardized mortality ratio (SMR) is a ratio between observed number of deaths in a study population and the number of expected deaths in the general population, stratified by age and gender. SMR was calculated comparing mortality in the study population with data from Statistics Sweden [22]. The Mid-p exact test was used to calculate confidence intervals for SMR.

The Kaplan-Meier survival function was plotted for different age and gender groups, compared to data from the general population, matched by age and gender (Statistics Sweden). The results were considered statistically significant for *p*-values ≤0.05. The statistical software used was R.

### Characteristics of the study population

There were 3777 patients (67% males) with open tibial fractures admitted to Swedish hospitals during the study period. Most fractures were located in the tibial shaft (60%), 14% were located in the proximal part and 26% in the distal part of tibia. The mean age of the patients at admission was 47 (SD 20) years (males 42 [SD 20] and females 55 [SD 22]). The mean follow-up time was 6 (SD 3.8) years. Regarding surgical fixation methods, the most usual method was intramedullary nailing (32%). Subsequent methods were combinations of external fixation and other (22%), plating (9%), external fixation only (8%), closed reduction and cast (4%), whereas 26% were fixated using miscellaneous methods. Causes of accident were in descending order motor vehicle accident (MVA, 43%), fall from standing height (21%), miscellaneous (19%), fall from height (11%) and unspecified falls (6%).

## Results

### Mortality rate

Out of the population of 3777 patients, 425 individuals (212 males and 213 females) died during the study period. This resulted in a total mortality rate of 11% during the study period of 13 years. We excluded 11 male patients from the analysis because of incomplete data. The primary outcome, mortality in the population after 90 days was 2% (66 patients). Mortality from 90 days to 2 years was 3% (109 patients). These numbers varied across age-groups, with the highest mortality being 11% after 2 years in patients aged 65 years and older (Table 1).

**Table 1:** Mean length of stay and mortality rates in different age groups

Age [years]	15–39	40–64	65–101
Number of patients [n]	1456	1564	744
Number of deaths [n]	28	111	286
90-day mortality [n (%)]	6 (0.4)	11 (0.7)	49 (6.5)
90-day to 2-year mortality [n (%)]	8 (0.6)	24 (1.6)	77 (11.1)
Mortality during entire study period [%]	1.9	7.6	38.4

### Length of stay

Mean LOS for primary admission ranged from 9 days (SD 15) in younger patients, to 10 days (SD 12) in middle-aged, and 14 days (SD 16) among the oldest.

### Causes of death

**Fig. 1:**
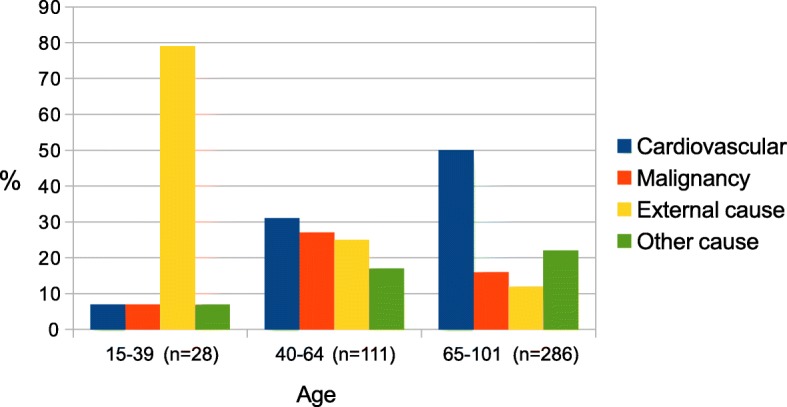
Causes of death in different age groups

Death was predominantly caused by cardiovascular disorders (180 deaths, 42%), malignant neoplasms (79 deaths, 19%) and by external causes (83 deaths, 20%) (Fig. 1). A complete list of all causes of death is shown in the Appendix.

Among younger patients with an age of 15–39 years, the dominating cause of death was external (79%). In the elderly (≥65) external causes accounted for only 12% of the deaths. These proportions were similar in men and women. Death causes in different age groups are shown in Fig. 1. A transport accident (35 cases out of all 425 deaths) was the most common external cause. Less frequent were suicides (*n* = 12), poisoning (*n* = 10), falls (*n* = 8), homicides (*n* = 3), drowning (*n* = 2) and miscellaneous (*n* = 13).

**Fig. 2:**
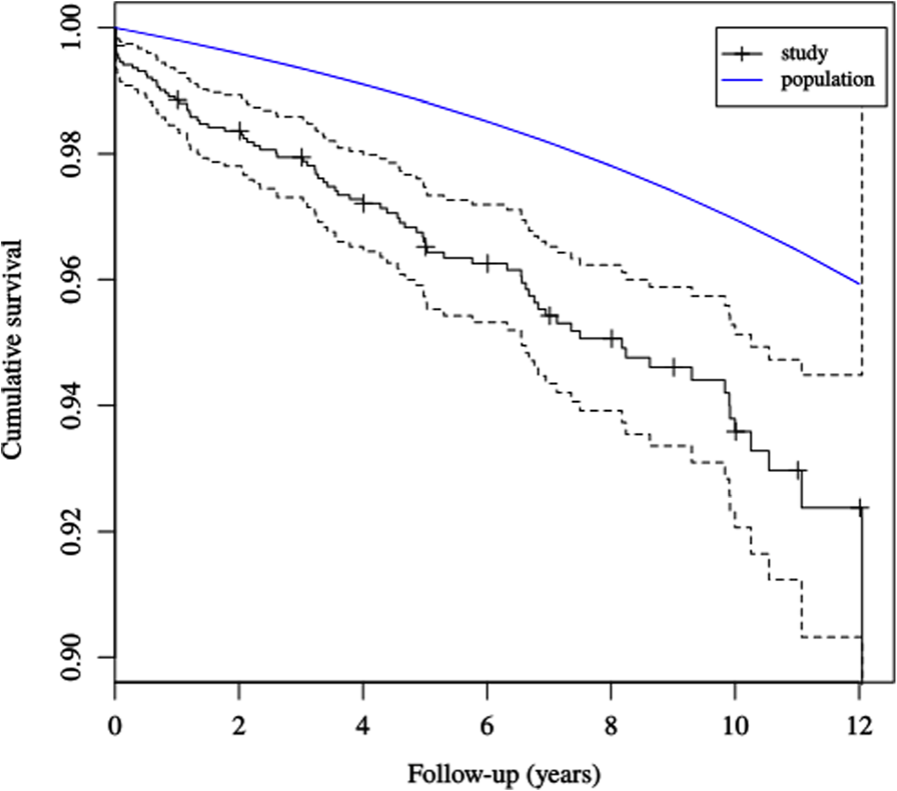
Kaplan-Meier survival function with 95% confidence interval for males aged 15-60 years compared to the general population of Sweden

We looked further at the subgroup of males aged 15–60 years as males are specifically exposed to traumatic injuries [1, 2] (Fig. 2). Twelve individuals died within 3 months after they sustained their open tibial fracture, all because of the initial trauma or its complications. Six of them died on the day of the accident. All individuals had sustained severe high-energy trauma like motor cycle accidents, pedestrians hit by car, blast injury or a helicopter accident. Among those who died between 3 and 12 months (*n* = 10), 4 deaths were caused by malignancies or co-morbidities and 6 deaths were due to external causes.

### Standard mortality ratio (SMR)

Standard Mortality Ratio (SMR) and excess mortality in the different age groups was estimated at 0–90 days and 90 days–2 years (Tables 2, 3). There was an overall increased excess death rate, although in some groups there were very few individuals. SMR was higher for early mortality (Table 4).

**Table 2:** Standard Mortality Ratio (SMR) and excess death rate until 90 days after injury

Age	Gender	Person-years	Deaths	SMR (95%-CI)	Expected deaths	Excess deaths	Excess death rate
15–39	males	286	5	28 (10–62)	0.178	4.822	1686
	females	79	2	80 (13–264)	0.025	1.98	2532
40–64	males	270	8	6.8 (3.2–13)	1.17	6.83	2527
	females	121	3	6.8 (1.7–19)	0.44	2.56	2119
65+	males	75	25	13 (9–19)	1.88	23.12	30,824
	females	111	26	22.6 (15–74)	1.15	24.85	22,387

SMR and excess death rate in different age and gender groups until 90 days after injury. *CI* confidence interval

**Table 3:** Standard Mortality Ratio (SMR) and excess death rate from 90 days until 2 years

Age	Gender	Person years	Deaths	SMR (95%-CI)	Expected deaths	Excess deaths	Excess death rate
15–39	males	2220	6	4.8 (2–10)	1.25	4.75	237
	females	546	2	11.8 (2–39)	0.17	1.83	335
40–64	males	270	8	6.8 (3–13)	1.17	6.83	2527
	females	845	5	1.6 (0.6–3.6)	3.09	1.91	227
65+	males	525	25	1.9 (1.3–2.8)	13.17	11.83	2252
	females	777	55	6.8 (5–9)	8.05	46.95	6 04

SMR and excess death rate in different age and gender groups from 90 days until 2 years after injury. *CI* confidence interval

**Table 4:** Risk factors for death within 90 days of the injury

	Crude			Adjusted		
	OR	95%-CI	*p*	OR	95%-CI	*p*
Gender						
Males	1.0	ref		1.0	ref	
Females	1.84	1.14–2.98	0.01	0.84	0.48–1.46	0.55
Age						
15–39	1.0	ref		1.0	ref	
40–64	1.54	0.61–4.20	0.37	1.56	0.61–4.31	0.36
65–101	23.3	11.2–56.6	< 0.005	25.7	11.8–64.8	< 0.001
Amputation						
No	1.0	ref		1.0	ref	
Yes	6.28	2.69–12.94	< 0.005	4.8	1.9–11.1	< 0.001
Length of stay						
Unit per extra day	1.00	0.97–1.02	0.89	0.98	0.95–1.00	0.07
Mechanism of injury						
Fall from standing height (*n* = 20)	1.0	ref		1.0	ref	
Fall from height (*n* = 15)	0.03	0.019–0.045	0.76	1.05	0.51–2.13	0.90
Unprotected road-users (*n* = 14)	0.46	0.22–0.91	0.026	0.83	0.38–1.76	0.63
Car crash (*n* = 7)	0.77	0.30–1.76	0.56	1.65	0.61–4.06	0.29
Other vehicles/objects (*n* = 5)	0.42	0.14–1.05	0.087	0.74	0.23–2.01	0.58
Self-destructive injury (n = 2)	0.75	0.12–2.63	0.70	1.64	0.25–6.39	0.53
Else (*n* = 6)	0.69	0.25–1.64	0.43	0.68	0.23–1.71	0.44

*OR* odds ratio*, CI* confidence interval*, ref* reference value (adjusted for sex, age, amputation, length of stay (LOS) and mechanism of injury)*, p p*-value*, n* number of patients

Logistic Regression Analysis and Cox Regression Analysis of Risk factors

Logistic regression analysis revealed factors associated with death during the first 90 days. For early death, adjusted analysis showed that factors that elevated the risk for death were age 65 and above and limb amputation (Table 4). Cox regression analysis revealed risk factors for death from 90 days and beyond. For later death, the independent risk factors were age 40 and above, limb amputation, increasing length of stay and cause of accident. Fall from standing height was chosen as reference variable among causes of accidents, regarded as a low energy accident. Among later deaths, a fall from standing height was associated with a higher mortality (Table 5).

**Table 5:** Risk factors for death from 90 days and beyond

	Crude			Adjusted		
	HR	95%-CI	*p*	HR	95%-CI	*p*
Gender						
Men	1.0	ref		1.0	ref	
Women	2.39	1.95–2.94	0.005	0.94	0.75–1.17	0.58
Age						
15–39	1.0	ref		1.0	ref	
40–64	4.28	2.72–6.74	< 0.005	3.67	2.32–5.81	< 0.001
65–101	31.1	20.3–47.8	< 0.005	22.9	14.7–35.7	< 0.001
Amputation						
No	1.0	ref		1.0	ref	
Yes	2.6	1.60–4.23	< 0.005	1.79	1.08–2.95	0.02
Length of stay						
Unit per extra day	1.011	1.009–1.014	< 0.005	1.01	1.009–1.017	< 0.001
Mechanism of injury						
Fall from standing height (*n* = 66)	1.0	ref		1.0	ref	
Fall from height (*n* = 19)	0.51	0.38–0.67	< 0.005	0.59	0.44–0.78	< 0.001
Unprotected road users (*n* = 8)	0.21	0.15–0.28	< 0.005	0.34	0.25–0.48	< 0.001
Car crash (*n* = 2)	0.21	0.13–0.36	< 0.005	0.36	0.21–0.62	< 0.001
Other vehicles/objects (*n* = 4)	0.17	0.10–0.29	< 0.005	0.30	0.18–0.51	< 0.001
Self-destructive injury (*n* = 3)	0.40	0.18–0.91	0.029	1.20	0.53–2.75	0.66
Else (*n* = 10)	0.43	0.29–0.63	< 0.005	0.48	0.32–0.72	< 0.001

*HR* hazard ratio, *CI* confidence interval, *ref* reference value (adjusted for sex, age, amputation, length of stay (LOS) and mechanism of injury), *p p*-value, *n* number of patients

## Discussion

In this nationwide study of all Swedish patients with open tibial fractures from 1998 to 2010 we found an increase in mortality compared to the general population. Younger males seem to be a subgroup of patients prone to violent death and self-destructive behaviour.

### Mortality and SMR

Overall mortality after 90 days was 2%. This number is relatively low but pronouncedly higher than in the population, showed by an increased SMR. In fact, SMR was increased in all age groups, men and women, showing that the number of observed deaths exceeded the number of expected deaths in the population.

Connelly et al. conducted a study of 1474 patients with tibial fractures, closed as well as open, with a long-time follow-up of 12–22 years [15]. The mortality rate in patients over the age of 75 years was 42%. In our study, total mortality during the study period of 13 years was 38% among patients 65–101 years. These results are similar and also demographic data indicate a comparable population. In a small prospective series of 54 elderly patients (> 65 years) with tibial fractures the mortality rate was 11% after 6-months follow-up [12]. No statistical significant difference was found between open and closed fractures, in contrast to two other studies [13, 14]. Clement et al. studied the outcome of 225 elderly patients with tibia fractures during a 9 year period and found a significantly higher mortality in patients with open fractures compared with patients with closed fractures (27% vs 17%, *p* < 0.001) [13]. They also found an increased SMR.

Elderly patients are known to have an increased mortality after injuries. This has been shown in hip fractures [9, 23, 24], tibial fractures [12–14], open ankle fractures [25] and polytraumatized elderly patients [26, 27]. Our study supports this. Excess death rate was markedly increased in the elderly. A great focus in Swedish healthcare has been on hip fracture patients but in our opinion there is a need to prioritize all elderly patients. The impact of orthogeriatricians competency was studied in hip fracture patients [28]. In this study, they found that the number of orthogeriatricians hours per patient had an effect to reduce mortality regardless of time to surgery.

Causes of death were extracted from registry data. The reliability of the underlying cause of death was 77% according to a study from 1995 [29]. Younger age, malignancies and acute diseases were associated with higher reliability. In our study, death was predominantly caused by cardiovascular and respiratory disorders (180 deaths, accounting for 42%), malignant neoplasms (79 deaths, 19%) and by external causes (83 deaths, 20%). External causes included accidents as well as self-destructive behaviour. As a comparison, in 2005 the dominating causes of death in the Swedish population were cardiovascular (42%) and malignant neoplasms (25%), whereas injuries and poisoning was the cause in only 5% of the cases [30]. In our study, external causes were overrepresented in all age groups and by far the most frequent cause among the youngest. This is in most cases explained by the accident itself as leading to death. Among elderly patients still cardiovascular disease was the most common cause of death. This sub-group of elderly patients sustain preferably low energy accidents that mostly will not lead directly to death. However, they are likely to have more co-morbidities that will be risk factors for cardiovascular causes of death.

Kaplan-Meier survival curves for younger males showed the increased mortality rate that was particularly increased during the first 2 years after the injury. Most individuals died relatively soon after their open fracture and apparently the cause of death was linked to the injury and its complications. Mortality rate continued to be increased even after the first months and we interpreted this as a subgroup of patients prone to dying of external causes. Causes of death often indicated violent accidents and self-destructive behaviour among these patients. This is consistent with work by Giannoudis et al., which demonstrated more problems with ongoing anxiety and depression in patient with Gustilo-type IIIB and IIIC fractures compared with the group of closed tibial fractures [6]. This fact raises the need for interventional measures such as psychosocial support after traumatic injuries and suicide prevention. Causes of accident were also analysed as independent risk factors for death. In our study, other causes of accident than a fall on the same plane were less associated with death. Our interpretation of this is that patients with co-morbidities and frailty are prone to sustain these injuries by low-energy accidents. Unprotected road-users as cyclists and motor bike drivers were shown to be at risk for fatal accidents in another study from France [31]. In our study the number of individuals in those sub-groups was small, and a statistical association may be difficult to show. Notably, in Sweden there is a national goal of no deaths in traffic including work on separate roads for cyclists.

Among the early deaths, a short LOS was associated with a higher risk of death. This illustrates the fact that many early deaths occur during the first few days after the accident.

On the other hand, among later deaths, a longer LOS was associated with increased risk of death. A probable explanation could be that complications and co-morbidities lead to longer time in hospital. We had no knowledge of co-morbidities in this set-up which is a limitation of the study. In the Swedish patient register, primary diagnosis has a high validity but for co-morbidities data are more unsecure [16]. Similar conclusions were drawn in another study from the Unites States [32]. Considering this, we chose not to perform statistical analyses on co-morbidities.

Even though this is a nationwide study of the entire Swedish population of patients with open tibial fractures, the number of deaths was low. There were subgroups with very few patients. Statistical associations may be difficult to show.

This is a registry study with its advantages and disadvantages. Classifications like ASA (American Society of Anesthesiologists) class and ISS (Injury Severity Score), that would have been of interest in analysis, are not available from the register. Furthermore, there is no registration of the severity of the injury such as Gustilo classification. More severe injuries have higher risk for complications, as infection and amputation, and this might influence the risk of death [33–35].

## Conclusion

Absolute mortality after open tibial fracture is relatively low, 2% after 90 days and 3% after 2 years, although the risk of death is increased for all age and gender groups compared with the Swedish general population. External causes of death are over-represented and indicates a subgroup with a risky behaviour among younger males.

Patients aged 65 years and above have a pronouncedly enhanced excess death rate after open tibial fracture and should be treated with priority. Geriatric units and/or specialized geriatric care for elderly patients with traumatic injuries would be of value.

## Appendix

**Table 6:** Death causes according to the European Shortlist in males and females in the different age groups

Death causes	All		Age					
			15–39		40–64		65+	
	Male	Female	Male	Female	Male	Female	Male	Female
Respiratory	3	16				2	3	14
Circulatory								
Ischemic heart disease	46	29	1		17	1	28	28
Cerebrovascular disease	9	25			3	2	6	23
Miscellaneous	18	34	1		7	2	10	32
Gastrointestinal	2	8			1	1	1	7
Mental disorders	9	13	1		1	2	7	11
Malignant neoplasms	46	33	2		20	10	24	23
Endocrine disorders	8	4		1	2		6	3
Musculoskeletal	1	1			1			1
Genitourinary		3						3
Diseases of the nervous system		8				2		6
Unknown	7	9			5		2	9
Miscellaneous	4	6			2	2	2	4
External causes								
Transport accident	28	7	7		9	2	12	5
Fall	4	4	1		1		2	4
Poisoning	6	4	4		1	4	1	
Suicide	8	4	6	1	1	2	1	1
Homicide	2	1	1	1	1			
Drowning	2		1		1			
Miscellaneous	9	4			6		2	5
Total	425		28		111		286	

## Abbreviations

ICD: International classification of diseases
LOS: Length of hospital stay
SMR: Standard mortality ratio
SNHDR: Swedish national hospital discharge register
